# Supplementary material for: Iterative improvement in the automatic modular design of robot swarms
Source: PeerJ Comput Sci. 2020 Dec 7;6:e322. doi: 10.7717/peerj-cs.322 (PMC7924708; doi:10.7717/peerj-cs.322)
Supplement: Supplemental Information 3 [file peerj-cs-06-322-s003.zip › argos3/doc/api/standalone/a00568_source.html]

ARGoS: plugins/simulator/entities/gripper\_equipped\_entity.cpp Source File


- Main Page
- Related Pages
- Namespaces
- Classes
- Files

- File List
- File Members

# plugins/simulator/entities/gripper\_equipped\_entity.cpp

Go to the documentation of this file.

```
00001 
00007 #include "gripper_equipped_entity.h"
00008 #include <argos3/core/simulator/space/space.h>
00009 
00010 namespace argos {
00011    
00012    /****************************************/
00013    /****************************************/
00014 
00015    CRange<Real> UNIT(0.0f, 1.0f);
00016 
00017    /****************************************/
00018    /****************************************/
00019 
00020    CGripperEquippedEntity::CGripperEquippedEntity(CComposableEntity* pc_parent) :
00021       CEntity(pc_parent),
00022       m_fLockState(0.0f),
00023       m_fLockThreshold(0.5f),
00024       m_pcGrippedEntity(NULL) {
00025       Disable();
00026    }
00027          
00028    /****************************************/
00029    /****************************************/
00030          
00031    CGripperEquippedEntity::CGripperEquippedEntity(CComposableEntity* pc_parent,
00032                                                   const std::string& str_id,
00033                                                   const CVector3& c_offset,
00034                                                   const CVector3& c_direction,
00035                                                   Real f_lock_threshold) :
00036       CEntity(pc_parent,
00037               str_id),
00038       m_cOffset(c_offset),
00039       m_cInitOffset(c_offset),
00040       m_cDirection(c_direction),
00041       m_cInitDirection(c_direction),
00042       m_fLockState(0.0f),
00043       m_fLockThreshold(f_lock_threshold),
00044       m_pcGrippedEntity(NULL) {
00045       Disable();
00046    }
00047          
00048    /****************************************/
00049    /****************************************/
00050 
00051    void CGripperEquippedEntity::Init(TConfigurationNode& t_tree) {
00052       try {
00053          /* Parse id */
00054          CEntity::Init(t_tree);
00055          /* Parse gripper attributes */
00056          GetNodeAttribute(t_tree, "offset", m_cOffset);
00057          m_cInitOffset = m_cOffset;
00058          GetNodeAttribute(t_tree, "direction", m_cDirection);
00059          m_cInitDirection = m_cDirection;
00060          GetNodeAttributeOrDefault(t_tree, "lock_threshold", m_fLockThreshold, m_fLockThreshold);
00061       }
00062       catch(CARGoSException& ex) {
00063          THROW_ARGOSEXCEPTION_NESTED("Error while initializing the gripper entity \"" << GetId() << "\"", ex);
00064       }
00065    }
00066          
00067    /****************************************/
00068    /****************************************/
00069 
00070    void CGripperEquippedEntity::Reset() {
00071       m_fLockState = 0.0f;
00072       m_cOffset = m_cInitOffset;
00073       m_cDirection = m_cInitDirection;
00074       ClearGrippedEntity();
00075    }
00076 
00077    /****************************************/
00078    /****************************************/
00079          
00080    void CGripperEquippedEntity::SetLockState(Real f_lock_state) {
00081       UNIT.TruncValue(f_lock_state);
00082       m_fLockState = f_lock_state;
00083    }
00084 
00085    /****************************************/
00086    /****************************************/
00087          
00088    void CGripperEquippedEntity::SetLockThreshold(Real f_lock_threshold) {
00089       UNIT.TruncValue(f_lock_threshold);
00090       m_fLockThreshold = f_lock_threshold;
00091    }
00092 
00093    /****************************************/
00094    /****************************************/
00095          
00096    CEmbodiedEntity& CGripperEquippedEntity::GetGrippedEntity() {
00097       if(m_pcGrippedEntity != NULL) {
00098          return *m_pcGrippedEntity;
00099       }
00100       else {
00101          THROW_ARGOSEXCEPTION("Entity \"" << GetId() << "\" is not gripping anything.");
00102       }
00103    }
00104 
00105    /****************************************/
00106    /****************************************/
00107          
00108    REGISTER_STANDARD_SPACE_OPERATIONS_ON_ENTITY(CGripperEquippedEntity);
00109 
00110    /****************************************/
00111    /****************************************/
00112 
00113 }
```

---

Generated on 10 Jul 2018 for ARGoS by 
 1.6.1
